# ^1^H, ^13^C, ^15^N backbone chemical shift assignment of P18ink4c from *Danio rerio* (zebrafish) using solution-state NMR spectroscopy

**DOI:** 10.1007/s12104-025-10245-2

**Published:** 2025-07-28

**Authors:** Aakriti Sethi, Pierre de Cordovez, Biswaranjan Mohanty, Vanessa K. Morris, Christoph Göbl

**Affiliations:** 1 Mātai Hāora - Centre for Redox Biology and Medicine, Department of Pathology and Biomedical Science, University of Otago, Christchurch, New Zealand; 2 Sydney Analytical Core Research Facility, The University of Sydney, Sydney, NSW Australia; 3 School of Biological Sciences, University of Canterbury, Christchurch, New Zealand; 4 Biomolecular Interaction Centre, University of Canterbury, Christchurch, New Zealand

**Keywords:** INK4 family of proteins, P18ink4c, *Danio rerio*, NMR resonance assignment, Ankyrin repeat protein

## Abstract

The INK4 family of proteins restricts uncontrolled cell cycle progression by inhibiting cyclin-dependent kinases 4 and 6. The family consists of small, monomeric and mainly alpha-helical proteins that are conserved across all vertebrate species. We recently discovered that the human INK4 protein p16 converts into amyloid structures upon oxidation of the single cysteine residue present. Here we investigate the *Danio rerio* (zebrafish) orthologue P18 protein. The 170-residue protein contains two cysteines which may similarly mediate transition into amyloids upon oxidation. We present the near complete backbone assignment of the reduced P18 protein in solution. These chemical shift data provide the foundation for studying oxidation-induced structural changes and protein interactions.

## Biological context

Eukaryotic cell division is a complex process that is tightly regulated throughout the different stages of the cell cycle (Hochegger et al. 2008). Several checkpoints ensure that each stage is completed before proceeding to the next step. One major checkpoint is the G1 to S transition which ensures the presence of growth signals and nutrients, the correct cell size and the lack of DNA damage (Hengstschläger et al. 1999; Heichman and Roberts 1994). It is tightly controlled by cyclin-dependent kinases 4 and 6 (CDK4/6), which form a complex with cyclin-D to phosphorylate the retinoblastoma protein to enter the S phase of the cell cycle (Malumbres and Barbacid 2009). CDK4/6 are inhibited by the family of INK4 proteins and its best characterised member is the human protein p16^INK4A^ (p16) (Serrano 1997). It is a 16.5 kDa small, all-α helical protein consisting of four sequential ankyrin repeats and it tightly binds to the kinase to block ATP interaction, thereby preventing cell cycle progression (Russo et al. 1998). p16 is therefore a major cell cycle regulator and is frequently mutated in various human cancers (Sherr 1996). Databases reveal a large number of mutations and several single-point variants have been shown to critically drive tumourigenesis through impaired ability to inhibit CDK4/6 (Tate et al. 2019).

We recently reported that p16 can undergo a major structural change. The oxidation of its single cysteine residue leads to the formation of a disulfide crosslinked dimer that subsequently folds into β-sheet-based amyloid fibrils (Göbl et al. 2020). Biochemical analysis suggests that amyloid p16 no longer inhibits CDK4/6 and therefore the alternative structural state can be considered a loss-of-function conformation (Heath et al. 2024a, b). Several single-point variants from human cancers showed a faster transition into amyloid structures upon oxidation. Interestingly, some rationally designed single-point variants remained stable monomers upon oxidation in contrast to the rapidly converting wild-type species. This raises the question of whether the p16 amyloid state has a functional role. Preliminary data shows that other human homologues and orthologues amongst different species can also transition into fibrillar structures upon cysteine oxidation.

Cell division lies at the interface of embryonic development, tumorigenesis, tissue regeneration, and ageing. *Danio rerio* (Zebrafish) are an excellent model organism to study these processes due to their genetic similarity to humans and their fast growth (Lieschke and Currie 2007). Here, we report the backbone chemical shift assignment of the ink4 family *D. rerio* protein P18ink4c (P18) under monomeric, reducing conditions. It harbours two cysteine residues and it is predicted to consist of five ankyrin repeats. No experimental structures of this protein have been reported yet but in the structural model, both cysteine residues are accessible in loop regions, suggesting their availability for oxidation. The chemical shift assignment will allow for studying of site-specific modifications and the oxidation-induced amyloid transition on a per-residue level and sets the basis for the solution structure determination of the monomeric conformation. Furthermore, studying the recombinant zebrafish P18 protein enables the combination of biochemical and NMR experiments with future in vivo studies in this vertebrate model.

## Methods and experiments

### Protein sample preparation

An *Escherichia coli* codon-optimised gene of *D. rerio* P18 L7I (Uniprot ID: B0UXZ0) was cloned into a pETZ2 vector with an N-terminal hexa-histidine tag followed by a Z-tag (protein A) and a TEV (tobacco etch virus) protease cleavage site by GenScript Biotech. Bacterial transformation for protein expression was carried out using *E. coli* BL21 (DE3) competent cells and these were plated on LB broth agar plates, including kanamycin as a selection marker. Uniformly labeled ^15^N and ^15^N, ^13^C protein samples were prepared using a protocol obtained from Dr. Motoshi Suzuki and Dr. Nico Tjandra (NIH, USA) and isotopes were purchased from Cambridge Isotope Laboratories, Inc. For this, a fresh 1 L batch of minimal media was prepared using 13 g KH_2_PO_4_, 10 g K_2_HPO_4_, 9 g Na_2_HPO_4_, 2.4 g K_2_SO_4_ at pH 7.2, 0.8 g ammonium chloride (^15^NH_4_Cl), 4 g glucose (unlabeled or ^13^C_6_H_12_O_6_), 0.5 g MgCl_2_ and 5 ml of a micro-salt solution consisting of 30 mg CaCl_2_⋅H_2_O, 30 mg FeSO_4_⋅7H_2_O, 5.8 mg MnCl_2_⋅4H_2_O, 4 mg CoCl_2_⋅6H_2_O, 1.5 mg ZnSO_4_⋅7H_2_O, 0.1 mg CuCl_2_⋅2H_2_O, 1.3 mg (NH_4_)_6_Mo_7_O_24_⋅4H_2_O, and 25 mg EDTA), and 50 µg/mL of kanamycin. Random colonies were selected from the transformed agar plates and inoculated in 100 ml of minimal media. The culture was grown at 28 °C with 180 rpm orbital shaking. Protein synthesis was initiated with 0.5 mM of isopropyl-1-thio-D-galactopyranoside (IPTG) once the optical density (OD) 600 of the bacterial culture reached 0.4–0.6 and the culture was incubated at 20 °C overnight. The P18 protein was purified after sonication, centrifugation and application to immobilized metal affinity chromatography (IMAC, Ni-NTA Agarose, Qiagen) followed by size-exclusion chromatography on a HiLoad 16/600 Superdex 75 pg column operated by an Äkta pure purification system (Cytiva). The fractions containing the protein were pooled and TEV protease (about 1:50 enzyme-to-protein ratio) was added and incubated overnight at 4 °C, followed by application to the nickel-affinity beads and collection of the flow-through fraction. The cleaved protein was treated with 2 mM TCEP (tris(2-carboxyethyl) phosphine) for 10 min at 4 °C and exchanged into a 50 mM sodium phosphate buffer (pH 6.5) containing 2 mM DTT (dithiothreitol) and 200 µM EDTA (ethylenediaminetetraacetic acid). The protein was then concentrated to 200 µM and stored at -80 °C until further use.

### NMR experiments

All NMR experiments were performed at 25 °C and this temperature was determined by testing long-term stability of the sample. Uniformly ^15^N-labeled or ^13^C, ^15^N-labeled protein samples at a concentration of ~ 200 µM were used for data acquisition and contained 7% D_2_O to provide a deuterium lock signal. Some spectra, including 2D ^1^H-^15^N HSQC, 3D HNCO and 3D ^15^N-edited [^1^H,^1^H]-NOESY-HSQC (NOE mixing time of 100 ms) were acquired on a Bruker Avance III 600 MHz spectrometer equipped with a 5 mm triple-resonance room temperature probe (Bruker BioSpin, GmbH, Rheinstetten, Germany) using standard Bruker pulse sequences and conventional uniform sampling protocols. Additional triple resonance spectra were acquired on a Bruker Avance III 800 MHz spectrometer equipped with a 5 mm cryogenic triple-resonance probe. The experiments included 3D HNCA, CBCA(CO)NH, HNCACB, HBHA(CO)NH, HNHA and a 3D ^15^N-edited [^1^H,^1^H]-NOESY-HSQC (NOE mixing time of 80 ms), to facilitate sequence-specific backbone resonance assignments. P18 at ~ 200 µM concentration was unstable over time, with visible precipitation observed after three days. To complete data collection, multiple freshly prepared samples were used. These samples were stored at 4 °C and transferred to 5 mm Shigemi tubes (Shigemi Inc., Japan) immediately before each experiment. To accelerate data acquisition within the limited stability window, non-uniform sampling (NUS) was employed. Sampling schedules for each experiment were generated using Poisson-gap sampling (Hyberts et al. 2010). NUS was employed with sampling densities ranging from 15 to 25%, using Nyquist grids of 60 complex points in the ^15^N dimension and 128 or 256 complex points in the second indirect dimension, depending on the experiment: HNCA (20%, ^15^N: 60 × ^13^C^α^: 128); CBCA(CO)NH (22%, ^15^N: 60 × ^13^C^β^: 128); HNCACB (15%, ^15^N:60 × ^13^C^αβ^: 128); HBHA(CO)NH (20%, ^15^N: 60 × ^1^H^αβ^: 128); HNHA (25%, ^1^H^α^: 128 × ^15^N: 60); and NOESY-HSQC (25%, ^15^N: 60 × ^1^H^noe^: 256). To enhance sensitivity in the 3D HNCACB experiment, we employed a higher number of scans while decreasing the sampling density compared to other experiments. All NMR data, including 3D NUS data, were processed using NMRPipe (Delaglio et al. 1995). The spectra were analysed using CcpNmr Analysis version 3 (Skinner et al. 2016). The TALOS+ webserver was used to determine secondary structure predictions (accessed on 15th May 2025) (Shen et al. 2009). Further details of the specific experiments and sample conditions can be found in the BMRB deposition files (accession number 53200).

### Extent of assignment and data deposition

**Fig. 1:**
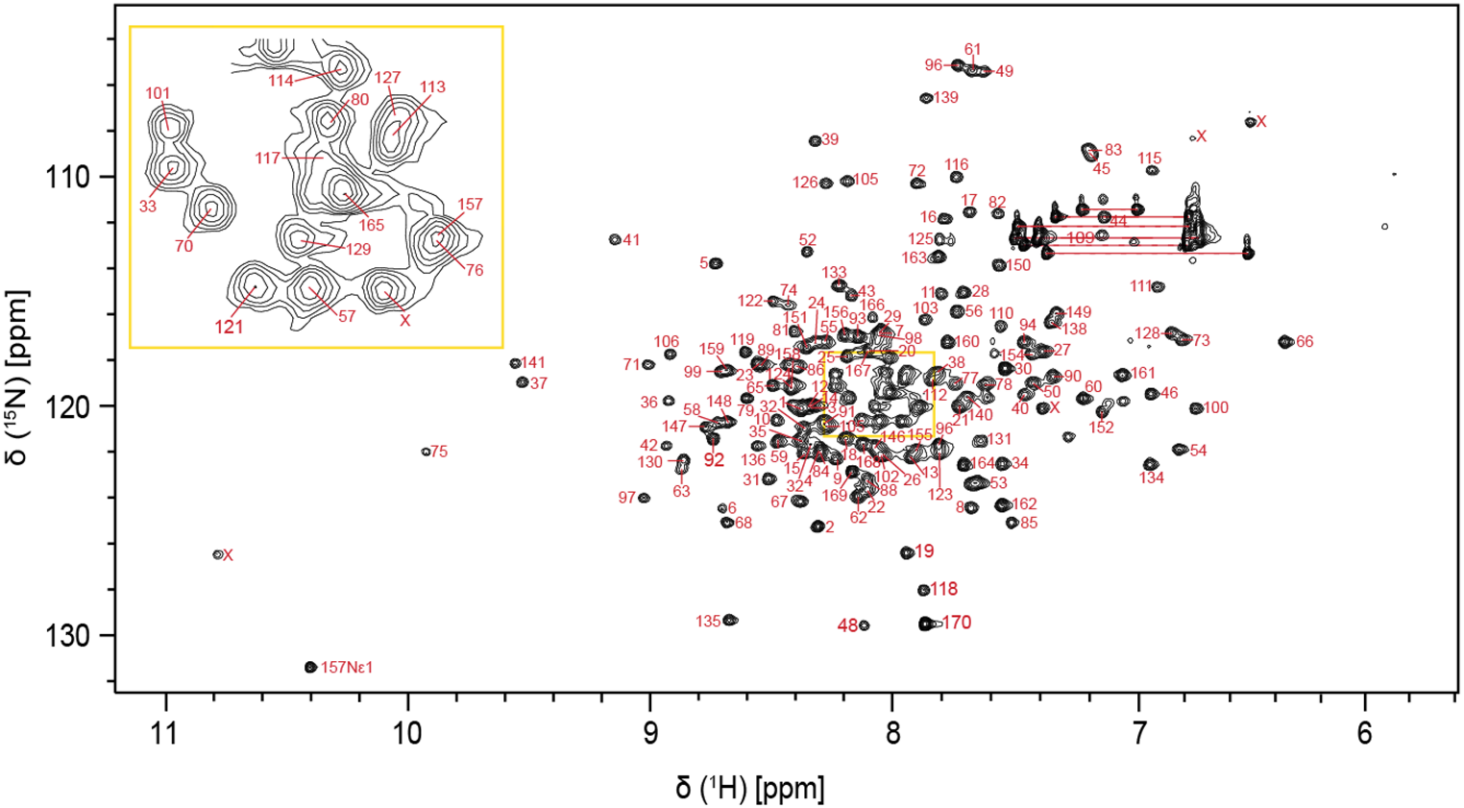
Two-dimensional ¹H-¹⁵N HSQC spectrum of P18 [1-170] at 25 °C at 600 MHz. Assigned peaks are labeled by their residue number. Asparagine and Glutamine amide side chains are connected by a horizontal red line and unassigned peaks are marked as ‘X’. Some of the unassigned peaks could potentially be aliased signals from the guanidinium group of Arginine side chains. The crowded region in the middle is magnified in the yellow, boxed area

The P18 protein comprises 170 residues and has a molecular weight of 18.7 kDa. The amino acid numbering is based on the UniProtKB database entry B0UXZ0. We sequenced the *cdkn2c* gene from our laboratory zebrafish and identified a missense mutation compared to the database, leading to the substitution of leucine in the 7th position with isoleucine. We included this mutation in our construct (L7I); it is otherwise identical to the database entry. The two-dimensional ¹H-¹⁵N HSQC spectrum shows well-resolved peaks, indicating a well-folded and structurally stable protein (Fig. 1). Using a standard protein assignment approach, a total of 156 non-proline residues (95.1%) of the backbone amides were assigned, excluding the two residues resulting from the TEV cleavage site at the N-terminus. K47, T87, L107, A137, R143, D144, L145 and R153 residues could not be confidently assigned due to either missing signals or severe peak overlaps across multiple dimensions. Additionally, combining the 3D HNCA, CBCA(CO)NH and HNCACB, we could determine the ^13^C^α^ and ^13^C^β^ chemical shifts for 99.4% and 98.7% of the assigned residues, respectively. The assignment was also guided by sequential HBHACONH and HNHA proton spectra. The 3D ^15^N-edited [^1^H,^1^H]-NOESY-HSQC spectrum further provided sequential NOEs to confirm the correct assignments. The chemical shift data of all the assigned residues is deposited under the BMRB repository catalogued under accession number 53200.

**Fig. 2:**
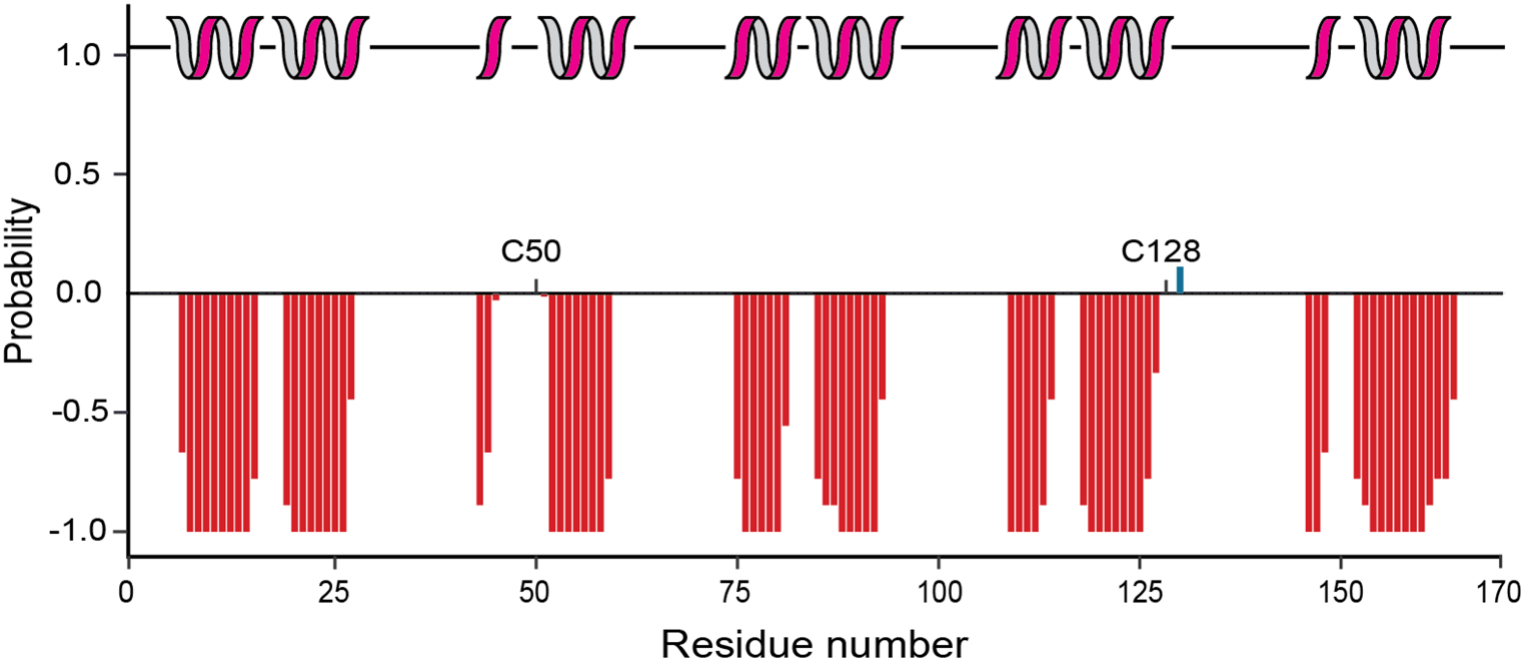
Secondary structure analysis of the assigned *D. rerio* P18 protein using the TALOS+ web server. Probability of formation of an α-helix (negative, red) or β-strand (positive, blue) is represented in red or blue bars, respectively. Cartoon representation of the predicted secondary structure is displayed at the top. α-helices are represented in magenta and the black solid line indicates loop regions. The positions of the cysteine residues are highlighted

The chemical shift data of the assigned residues was used to predict the secondary structure of the protein using the TALOS+ web server. The analysis indicated that P18 is mainly α-helical and the secondary structure is consistent with 5 ankyrin repeats. The first helix in the second ankyrin repeat comprises only three amino acids (Fig. 2). It is next to C50 present in a loop region, suggesting that the short helix could be functionally relevant. Notably, the structural arrangement of the second ankyrin repeat in P18 is similar to human p16, also harbouring a shorter helix, even when in complex with CDK6 (Russo et al. 1998). Also, the first helix of the fifth ankyrin repeat is rather short according to the secondary chemical shifts, however, four preceding amino acids are unassigned and homologues structures such as human p18 show a longer and typical α-helical structure in this region (Li et al. 1999). The second cysteine residue C128 is also present in a predicted loop region, and likely also available for oxidation. As observed for other ankyrin repeat proteins, the loop regions connecting the helix repeats are long and do not show a tendency for beta-sheet secondary structure elements.

## Data Availability

The chemical shift data of all the assigned residues is deposited under the BMRB repository catalogued under accession number 53200.
